# Inequities in Waiting Times for Major Elective Surgery Before and After the COVID-19 Pandemic: Socioeconomic and Sex Differences in the Southern Barcelona Metropolitan Area

**DOI:** 10.3390/healthcare14050571

**Published:** 2026-02-25

**Authors:** Carles Pericas, Carles Vilaplana-Carnerero, Violeta Poltorak, Ana Redondo, Cristina Masuet-Aumatell, Alba Tor-Roca, Constança Pagès-Fernández, María Grau

**Affiliations:** 1 Department of Medicine, University of Barcelona (UB), 08007 Barcelona, Spain; carles_vilaplana@ub.edu (C.V.-C.); albator@ub.edu (A.T.-R.); mariagrau@ub.edu (M.G.); 2 Epidemiology Service, Public Health Agency of Barcelona (ASPB), 08023 Barcelona, Spain; 3 Biomedical Research Consortium in Epidemiology and Public Health (CIBERESP), 28029 Madrid, Spain; 4 Sant Pau Research Institute (IR Sant Pau), 08041 Barcelona, Spain; 5 Service for the Promotion of Quality and Bioethics, Directorate-General for Health Planning and Regulation, Department of Health, 08028 Barcelona, Spain; 6 Department of Preventive Medicine, Hospital Universitari de Bellvitge, 08907 Barcelona, Spain; vpoltorak@bellvitgehospital.cat (V.P.); aredondon@gencat.cat (A.R.); cmasuet@bellvitgehospital.cat (C.M.-A.); 7 Bellvitge Biomedical Research Institute (IDIBELL), L’Hospitalet de Llobregat, 08907 Barcelona, Spain; 8 Subdirectorate-General for Medical Assessments, Directorate-General for Health Planning and Regulation, Department of Health, 08028 Barcelona, Spain; 9 Fundació Institut Universitari per a la Recerca a l’Atenció Primària de Salut Jordi Gol i Gurina (IDIAPJGol), 08007 Barcelona, Spain; 10 Research Institute for Nutrition and Food Safety (INSA-UB), 08921 Santa Coloma de Gramenet, Spain; 11 School of Pharmacy, University of Barcelona (UB), 08007 Barcelona, Spain

**Keywords:** waiting times, socioeconomic inequalities, elective surgery

## Abstract

**Background**: Waiting times for elective surgery are widely used as indicators of health system performance, particularly following the disruption caused by COVID-19. The objective of this study is to compare the waiting times for major elective surgeries in the Southern Barcelona Metropolitan Area before (2018–2019) and after (2022–2023) the pandemic, examining differences according to sociodemographic characteristics. **Methods**: A retrospective comparative study was conducted using data from the Southern Barcelona Metropolitan Area between 2018 and 2023. Adults registered on the waiting list for major elective surgical procedures were included. All analyses were stratified by sex. A stratified pre–post pandemic analysis was conducted to examine differences in waiting times by Socioeconomic Index. Waiting times were modelled using generalized linear models with a Gamma distribution and log link, adjusting for age and Socioeconomic Index quartiles. Interaction between period and Socioeconomic Index was tested. **Results**: The analysis included 73,055 individuals (50.5% women). Median waiting time decreased after the COVID-19 pandemic for both sexes (women: 128 to 121 days; men: 99 to 94 days). This reduction showed an inverse socioeconomic gradient. Adjusted analyses showed longer waits in the lowest versus highest Socioeconomic Index quartile after the pandemic (women: RR = 1.23; men: RR = 1.30). **Conclusions**: Waiting times for major elective surgery decreased after COVID-19. An exclusive focus on waiting time indicators may conceal structural barriers to access and contribute to inequalities. Equity-sensitive monitoring of elective care is essential to ensure a fair recovery of surgical services.

## 1. Introduction

Social inequalities in health are a key component when analysing access to and use of healthcare resources. The social structure, shaped by factors such as socioeconomic status, country of birth, gender, age, or working conditions, affects both the potential exposure to risks and the capacity of people to access health services and information [1,2]. The healthcare system can act as a social determinant of health and at the same time intensify previously existing inequalities [3,4]. Waiting lists serve as a critical indicator of equity, since they reflect both the pressure on the system and the inequalities in the ability to access care among different social groups [5,6].

Before the COVID-19 pandemic, the Spanish healthcare system was already going through a sustained process of structural tension. Spain operates a tax-funded system that provides universal coverage to the resident population. Major elective surgical procedures performed in public hospitals are fully covered, with no out-of-pocket payments for the surgical intervention itself. Alongside this universal public system, voluntary private health insurance exists and plays a complementary role, particularly in metropolitan areas, where it may provide alternative pathways to elective surgery through private providers. Despite universal entitlement, the public system was characterised by chronic underfunding, reductions in human resources, and increasing overload, especially in primary care and hospital services [7]. This pressure was particularly visible in metropolitan areas, where ageing populations, territorial inequalities, and high healthcare demand contributed to growing surgical waiting lists [8,9,10,11].

In this context, waiting times were not distributed homogeneously: several studies indicate that people with lower socioeconomic status and those living in more disadvantaged areas face greater difficulties in accessing specialised procedures within the usual clinically appropriate timeframes [12,13].

The pandemic introduced a turning point. The hospital reorganisation needed to respond to the health emergency led to the massive cancellation of elective procedures, the reassignment of operating theatres and professionals to the care of COVID-19 patients, and a sudden decrease in referrals from primary care. International estimates indicate that tens of millions of elective surgeries were cancelled during the first wave, and Spain was no exception [14,15]. The accumulated effect of these interruptions resulted in an increase in waiting times with a marked impact in territories with high healthcare pressure and in those population groups already situated in more vulnerable positions [16,17,18,19,20].

COVID-19 not only disrupted healthcare circuits but also widened pre-existing social inequalities. Factors such as labour instability, the precarity of certain sectors, and territorial inequalities in the distribution of healthcare resources have contributed to creating an even deeper gap in access to specialised care [21,22]. In urban contexts like Barcelona (Spain), a more pronounced deterioration has been observed among groups with lower socioeconomic positions, consolidating a pattern that reflects how the social and health effects of the pandemic reinforced one another [4,23].

This interaction between social inequalities and healthcare pressure is especially relevant in the case of major elective surgeries, where delays can lead to functional deterioration, worsening symptoms, and emotional distress [24,25]. In metropolitan settings, increases in waiting times may reflect both structural limitations and differentiated social or administrative barriers [26]. Studying this evolution offers an opportunity to understand how organisational changes induced by the pandemic have affected access to surgical care based on different sociodemographic features.

The objective of the present study is to compare the evolution of waiting times for major elective surgeries in the Southern Barcelona Metropolitan Area before (2018–2019) and after (2022–2023) the pandemic, examining differences according to sociodemographic characteristics.

## 2. Materials and Methods

### 2.1. Design and Participants

We conducted a retrospective comparative study using administrative data from the Bellvitge University Hospital (L’Hospitalet de Llobregat), a tertiary referral hospital serving a defined catchment population within the Southern Barcelona Metropolitan Area. Two time periods were analysed: 2018–2019 (pre-pandemic) and 2022–2023 (post-pandemic). The years 2020–2021 were excluded as they corresponded to the acute phase of healthcare disruption during the COVID-19 emergency, characterised by widespread suspension of elective surgery and unstable waiting list dynamics. The study population included adults (≥18 years) residing in the hospital’s reference area and registered on the waiting list for major elective procedures during these periods. The registry analysed includes only publicly funded elective surgical activity. Individual private insurance status and private-sector utilisation are not available in the administrative data. This study was reviewed and approved by the Bioethics Commission of the University of Barcelona (IRB00003099) and the Bellvitge Hospital Ethics Committee (PR140/25).

### 2.2. Variables

Waiting time was calculated as the number of days between the date of inclusion on the surgical waiting list and the recorded date of surgery. Only procedures that were completed and had both a registered waiting list entry date and a recorded surgery date were included in the analysis. The registry does not provide information on cancellations or interim rescheduling processes prior to the final recorded surgery date. Sociodemographic characteristics included sex assigned at birth (woman or man), age, most common elective procedures, and primary care area of adscription (PCA), all obtained from the hospital database. Additionally, the mean Socioeconomic Index (Territorial Socioeconomic Index, TSI) for each PCA was retrieved from the Catalan Government’s open data portal and assigned to each participant. This composite indicator is based on standardised indicators of income, educational attainment, and employment, aggregated at small geographic units and scaled to a reference value of 100 for Catalonia. Higher values of the Socioeconomic Index indicate higher socioeconomic position [27]. For analytical purposes, the Socioeconomic Index was categorised into quartiles (Q1–Q4) based on its distribution in the study population. The cut-off values were as follows: Q1 ≤ [96.5], Q2 > [96.5]–≤[103.3], Q3 > [103.3]–≤[105.0], and Q4 > [105.0].

### 2.3. Statistical Analysis

Descriptive analyses were stratified by sex. Continuous variables were summarised as means and standard deviations (SD), and categorical variables as frequencies and proportions. Waiting time was described using median and interquartile range (IQR). Student’s *t*-tests, Mann–Whitney U tests, and chi-square tests were used to compare pre-pandemic vs. post-pandemic means, medians, and proportions, respectively.

To model waiting time (in days), we fitted generalized linear models (GLM) with a Gamma distribution and log link, suitable for strictly positive, right-skewed outcomes. All models were stratified by sex. The main exposure was the period indicator (pre- vs. post-pandemic), adjusted for age and Socioeconomic Index. Coefficients were exponentiated and reported as rate ratios (RR) with 95% confidence intervals (CI) and two-sided *p*-values. Interaction between period and Socioeconomic Index was assessed using likelihood ratio tests comparing nested models with and without the interaction term. When significant interaction was detected (*p* < 0.05), we performed stratified Gamma log-link models by period (pre-pandemic and post-pandemic), adjusting for age and modelling Socioeconomic Index using quartiles (Q), with Q4 (highest Socioeconomic Index quartile) as the reference category.

To assess the potential influence of changes in surgical case-mix across periods, we conducted a sensitivity analysis restricted to ophthalmological procedures, the most frequent elective surgery category in the dataset. The same analytical approach used in the main analysis was applied to this restricted sample, including sex-stratified models and adjustment for age and Socioeconomic Index.

All analyses were conducted using R (version 4.5.1; R Foundation for Statistical Computing, Vienna, Austria) [28].

## 3. Results

### 3.1. Descriptive Analysis

A total of 73,055 individuals were included in the analysis. Of these, 36,869 (50.5%) were women. Table 1 shows the characteristics of the sample stratified by sex and study period.

In the full sample, median waiting time for major elective surgeries decreased significantly for both sexes after the pandemic. Among women, median waiting time declined from 128 days (IQR: 43–230) in the pre-pandemic period to 121 days (IQR: 40–198) in the post-pandemic period (*p* < 0.001). Among men, waiting time decreased from 99 days (IQR: 36–214) to 94 days (IQR: 35–186) (*p* < 0.001) (Table 1).

The mean Socioeconomic Index did not change across periods among women, while a marginal statistically significant decrease was observed among men, most likely attributable to the large sample size. The distribution of surgery types differed significantly between periods for both sexes (*p* < 0.001), with a relative reduction in general surgery and changes in the proportional weight of other specialties across periods (Table 1).

**Figure 1:**
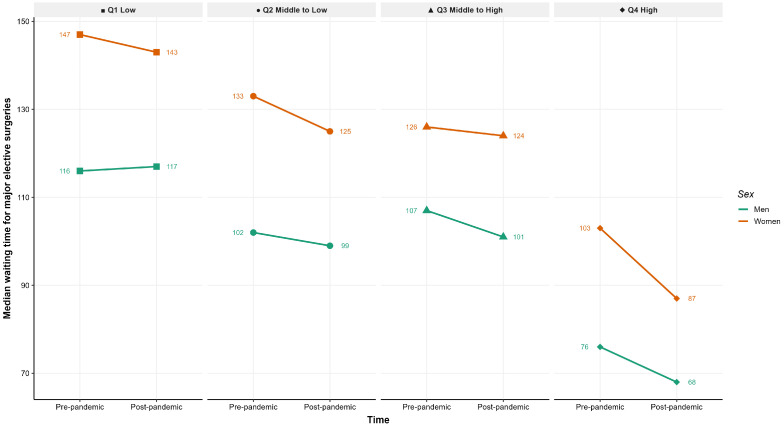
Median waiting time for major elective surgery before and after the COVID-19 pandemic, by socioeconomic Index quartile and sex.

Figure 1 shows a reduction in median waiting times in the post-pandemic period across all socioeconomic quartiles. Despite this overall decrease, a socioeconomic gradient persists, with longer waiting times in those with lower Socioeconomic Index both before and after the pandemic. Across all socioeconomic levels and periods, women consistently experienced longer waiting times than men.

### 3.2. Adjusted Analysis of Waiting Time

Table 2 presents the results from the sex-stratified Gamma regression models. After adjustment for age and Socioeconomic Index, the post-pandemic period was associated with a significantly shorter waiting time compared with the pre-pandemic period in both women (RR: 0.86; 95% CI: 0.84–0.88) and men (RR: 0.93; 95% CI: 0.91–0.95), equalling to a 14% and 7% reduction, respectively.

Age presented an inverse association with waiting time in both sexes, with shorter waiting times for older patients. Higher Socioeconomic Index values were associated with shorter waiting times for both women and men.

### 3.3. Interaction Between Time Period and Socioeconomic Level

A statistically significant interaction between pandemic period and Socioeconomic Index quartile was identified.

**Figure 2:**
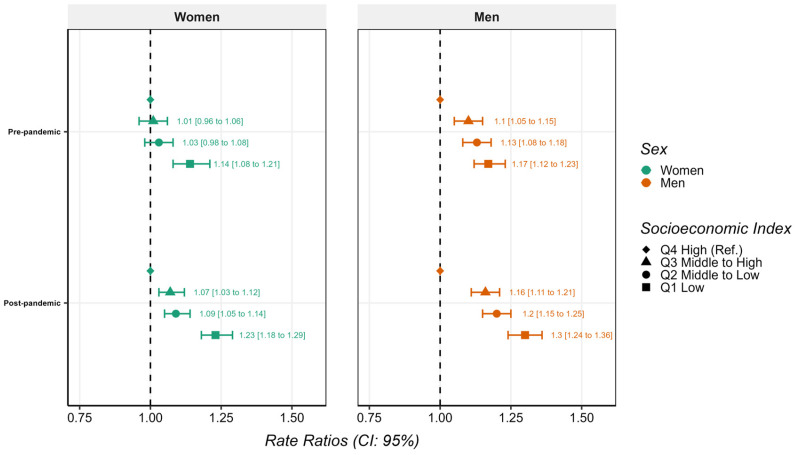
Association between Socioeconomic Index quartiles and waiting time for major elective surgery before and after the COVID-19 pandemic, stratified by sex. Estimates are shown as rate ratios (RR) with 95% confidence intervals from sex-stratified Gamma regression models adjusted for age. Q4 (highest quartile) was used as the reference category.

Figure 2 provides period-specific analyses stratified by Socioeconomic Index quartile and sex. Socioeconomic gradients in waiting time were observed in both periods, with longer waiting times among patients from groups with lower Socioeconomic Index.

Before the pandemic, women from the lowest Socioeconomic Index quartile showed significantly longer waiting times compared to the highest quartile (RR: 1.14; 95% CI: 1.08–1.21), while other socioeconomic groups did not present significant differences. In the post-pandemic period, all lower quartiles showed significantly longer waiting times if compared to the highest quartile, particularly among women in the lowest Socioeconomic Index quartile (RR: 1.23; 95% CI: 1.18–1.29).

Among men, in the pre-pandemic period, there were already progressively longer waiting times across decreasing Socioeconomic Index. These differences increased in magnitude after the pandemic, with men in the lowest Socioeconomic Index quartile experiencing a 30% longer waiting time compared to those in the highest quartile (RR: 1.30; 95% CI: 1.24–1.36).

### 3.4. Sensitivity Analysis

In the sensitivity analysis restricted to ophthalmological procedures (Supplementary Tables S1 and S2), median waiting times increased in the post-pandemic period compared with the pre-pandemic period. However, the socioeconomic gradient in waiting times persisted, with longer waits observed among lower Socioeconomic Index quartiles in both periods.

## 4. Discussion

The present study analysed changes in waiting times for major elective surgery in the Southern Metropolitan Area of Barcelona before and after the COVID-19 pandemic, focusing on Socioeconomic Index and sex differences. According to the obtained results, overall post-pandemic median waiting times were shorter when compared to the 2018–19 period. However, pre-existing socioeconomic inequalities in waiting time widened after the COVID-19 pandemic. This apparent paradox suggests that post-pandemic reductions in waiting time cannot be interpreted as clear evidence of generalised improvement in system performance and may instead be consistent with underlying structural dynamics affecting access to elective surgical care.

All these findings point to a system in which access to elective surgery might remain socially patterned, even within a universal healthcare framework [6]. These patterns also differed by sex, as women experienced longer waiting times overall and showed distinct evolving socioeconomic gradients before and after the pandemic, which may reflect broader gender-related social dynamics influencing socioeconomic position and access to care [13,29].

### 4.1. Socioeconomic and Gender Inequalities Before and After the Pandemic

The distinct waiting time trajectories for both women and men suggest potentially differentiated patterns in how socioeconomic inequalities in access to elective surgery evolved over time.

Consistent with previous research, our results show that women experienced longer waiting times for major elective surgery across both study periods [13]. However, prior to the pandemic, socioeconomic differences in waiting times among women appeared less pronounced than those observed among men. This pattern might relate to the interaction between gendered social roles and healthcare access, including factors such as informal support networks, which might temporarily attenuate socioeconomic gradients under relatively stable system conditions [29,30,31].

The emergence of a clearer socioeconomic gradient among women after the pandemic might be interpreted as an expression of the widening of social inequalities derived from the effect of COVID-19, more than an exclusive result from changes in healthcare systems and waiting lists organisation [32]. Growing evidence shows that the pandemic interacted with pre-existing social vulnerabilities, particularly in terms of formal and informal labour and economic stability, affecting more women in an unfavoured socioeconomic position [23,33,34]. This cumulative effect has been widely described in the literature as characteristic of a syndemic context, in which social determinants amplify both the risk and the indirect consequences of the health crisis [32].

A similar dynamic can also be observed among men, although with a different temporal manifestation. In this group, pre-existing socioeconomic gradients in waiting times were already present before the pandemic, and appeared more pronounced in the post-pandemic period. These findings indicate that, among men, the same syndemic processes might have intensified established inequalities rather than revealed previously attenuated differences.

Within this framework, the growing social difficulties in maintaining continuity of care, attending appointments, adapting to rescheduling, or sustaining a stable trajectory within the healthcare system are likely to have contributed to making socioeconomic differences more visible for both women and men [4,26,35].

### 4.2. Reduced Waiting Times in a Constrained System

A relevant finding of this study is that reductions in waiting times in the post-pandemic period coexist with apparently wider socioeconomic inequalities, a pattern previously described in health systems operating under sustained pressure [5]. It should be noted that the period analysed as post-pandemic (2022–2023) represents the initial stabilisation phase following the acute healthcare disruption of 2020–2021, rather than a fully consolidated recovery phase [16]. Longer follow-up would be required to assess sustained recovery trajectories in elective surgical care.

The interpretation of these findings must also consider the institutional characteristics of the Spanish healthcare system. Although Spain provides universal, tax-funded coverage and elective surgical procedures in public hospitals are fully covered, voluntary private health insurance plays a complementary role. In this context, widening socioeconomic gradients in public waiting times could partly reflect differential use of private sector surgical care among individuals with higher socioeconomic position [7]. However, as private insurance status and private-sector utilisation were not available in the dataset, this hypothesis cannot be directly tested.

This supports that shorter waiting times should not be interpreted as evidence of improved surgical capacity or efficiency of a health system [5]. Consistent with prior evidence, they might reflect increasing constraints earlier in the care pathway and result in more selective access to surgical waiting lists [26]. In this context, the population reaching the stage of being listed for surgery may represent a progressively less representative and more socially advantaged group of those in need of elective care. Reductions in waiting times among patients who are listed for elective surgery may reflect selective access mechanisms within the care pathway, changes in surgical case-mix across periods, or differential use of private-sector care rather than a genuine expansion of system capacity, an interpretation that aligns with prior evidence from health systems experiencing prolonged pressure [3,7].

### 4.3. Implications for Health-Related Policy

Some implications for health policy can be derived from the findings of this study, especially when addressing post-pandemic strategies for recovery. The results suggest that, while reducing surgical backlogs might be important, focusing exclusively on waiting times might entail neglecting structural barriers to access and indirectly exacerbating pre-existing inequalities [27,36].

Strengthening primary care should also be considered a central component of health systems recovery strategies [37]. Investments in staffing and organisational capacity in primary care are essential to ensure appropriate referral to specialised services [38]. Without addressing these shortcomings, measures aimed at increasing surgical throughput might mostly benefit individuals who are already better positioned to navigate the healthcare system [39].

Additionally, equity considerations should be applied to all waiting list management initiatives, especially in situations when capacity is constrained such as during the COVID-19 pandemic. Waiting times should be monitored using indicators disaggregated by socioeconomic position. This could help identify individuals and population groups at an increased risk of higher waiting times and support the incorporation of measures of social vulnerability together with clinical criteria [35,36]. The observed differences among sex also underline the importance of carrying out sex-disaggregated analyses, as differences in types of surgery, referral patterns, and gender roles can shape distinct access for women and men [29].

### 4.4. Limitations and Strengths

This study has some limitations. Firstly, socioeconomic position was measured using an area-level index, which may underestimate individual-level inequalities. Additionally, the analysis includes only individuals who were registered on the surgical waiting list and, therefore, cannot capture differences in unmet needs or in access prior to referral. This limitation is particularly relevant in the post-pandemic context and reinforces the need for integrated data systems that enable monitoring from primary care contact to surgical intervention. It’s important to note that private health insurance status and private sector surgical utilisation were not available in the dataset, preventing assessment of potential shifts between public and private care. Furthermore, the administrative waiting list registry did not include information on comorbidities, surgical complexity, diagnosis, referral pathways, or hospital capacity indicators (such as operating room availability, staffing levels, or cancellations). The absence of these variables prevents disentangling supply and demand mechanisms underlying the observed changes in waiting times. In this regard, a broader set of variables would have allowed for a more detailed exploration of the mechanisms that manifest into the observed inequalities. Additionally, the study was conducted within a single hospital catchment area in a large metropolitan setting, which may limit the generalisability of the findings to other regions or healthcare contexts.

However, some strengths should be acknowledged. The study uses population-based administrative data covering a large metropolitan area, applies statistical models appropriate for skewed waiting time distributions, and explicitly examines interactions between pandemic period and socioeconomic position. Stratification by sex also allows for a more nuanced understanding of inequalities that would not be present in pooled analyses.

## 5. Conclusions

In conclusion, this study shows that reductions in waiting times for major elective surgery in the post-COVID-19 period coexist with persistent and, in some instances, widening socioeconomic inequalities. The apparent improvements in aggregate waiting list indicators may mask underlying social gradients in access and should therefore be interpreted with caution. However, the present study cannot determine the specific mechanisms driving these patterns, as clinical prioritisation, referral trajectories, and system-level capacity indicators were not available.

The observed differences between sexes suggest that these dynamics do not operate uniformly across the population, with distinct socioeconomic gradients and trajectories for women and men that warrant further investigation. Within the context of a large metropolitan area in Spain and Catalonia, this highlights the need to reframe post-pandemic recovery strategies around equity. Addressing access barriers to timely elective procedures and structural determinants of health is crucial to avoid reinforcing social inequalities within universal healthcare systems.

## Figures and Tables

**Table 1:** Sociodemographic characteristics, waiting time, and type of elective surgery by sex and pandemic period.

	Women			Men		
	Pre-Pandemic (N = 18,278)	Post-Pandemic (N = 18,591)	*p*-Value	Pre-Pandemic (N = 18,299)	Post-Pandemic (N = 17,887)	*p*-Value
**Age (Years), Mean (SD)**	63 (16)	64 (16)	<0.001	62 (17)	64 (16)	<0.001
**Waiting days**	128.0 [43.0; 230.0]	121.0 [40.0; 198.0]	<0.001	99.0 [36.0; 214.0]	94.0 [35.0; 186.0]	<0.001
**Socioeconomic index**	101.3 (8.0)	101.3 (7.9)	0.504	101.3 (8.1)	101.2 (8.5)	0.003
**Type of surgery**			<0.001			<0.001
Cardiovascular	845 (4.6%)	778 (4.2%)		1506 (8.2%)	1389 (7.8%)	
General and Digestive	2305 (12.6%)	1099 (5.9%)		3449 (18.8%)	1677 (9.4%)	
Oral and Maxillofacial	414 (2.3%)	435 (2.3%)		431 (2.4%)	421 (2.3%)	
Orthopaedics and traumatology	3919 (21.4%)	3355 (18.0%)		2647 (14.5%)	2531 (14.1%)	
Plastic and Reconstructive	961 (5.3%)	1149 (6.1%)		566 (3.1%)	660 (3.7%)	
Thoracic Surgery	237 (1.3%)	230 (1.2%)		374 (2.0%)	350 (2.0%)	
Gynaecology	2382 (13.0%)	2403 (12.9%)		0 (0.0%)	0 (0.0%)	
Neurosurgery	384 (2.1%)	506 (2.7%)		354 (1.9%)	452 (2.5%)	
Ophthalmology	4806 (26.3%)	4844 (26.1%)		4081 (22.3%)	4006 (22.4%)	
Otorhinolaryngology	793 (4.3%)	891 (4.8%)		1268 (6.9%)	1160 (6.5%)	
Urology	727 (4.0%)	836 (4.5%)		3278 (17.9%)	2829 (15.8%)	
Other	505 (2.8%)	2065 (11.1%)		345 (1.9%)	2412 (13.5%)	

**Table 2:** Association between waiting time for major elective surgery and period indicator (pre- vs. post-pandemic), stratified by sex.

	Women			Men		
	RR	95% CI	*p*-Value	RR	95% CI	*p*-Value
Pre-post COVID-19	0.86	0.84; 0.88	<0.001	0.93	0.91; 0.95	<0.001
Age	0.99	0.99; 0.99	<0.001	0.99	0.99; 0.99	<0.001
Socioeconomic index	0.99	0.99; 0.99	<0.001	0.99	0.99; 0.99	<0.001

RR = rate ratio; CI = confidence interval.

## Data Availability

The data used in this study are administrative health records and are not publicly available due to ethical and legal restrictions related to data protection and patient confidentiality. Access to the data may be granted upon reasonable request to the corresponding author, subject to approval by the relevant data-holding authorities and ethics committees.

## Supplementary Materials

The following supporting information can be downloaded at: https://www.mdpi.com/article/10.3390/healthcare14050571/s1. Table S1: Association between waiting time for ophthalmology surgery and period indicator (pre- vs. post-pandemic), stratified by sex; Table S2: Association between Socioeconomic Index quartiles and waiting time for ophthalmology surgery before and after the COVID-19 pandemic, stratified by sex.

## Abbreviations

The following abbreviations are used in this manuscript:

CI	Confidence Interval
GLM	Generalised Linear Model
IQR	Interquartile Range
PCA	Primary Care Area
RR	Rate Ratio
SD	Standard Deviation
TSI	Territorial Socioeconomic Index
