# Supplementary material for: Inequities in Waiting Times for Major Elective Surgery Before and After the COVID-19 Pandemic: Socioeconomic and Sex Differences in the Southern Barcelona Metropolitan Area
Source: Healthcare (Basel). 2026 Feb 25;14(5):571. doi: 10.3390/healthcare14050571 (PMC12984575; doi:10.3390/healthcare14050571)
Supplement: Supplementary file 1 [file healthcare-14-00571-s001.zip › healthcare-4145107-supplementary.pdf]

Table S1: Association between waiting time for ophthalmology surgery and period indicator (pre- vs. post-pandemic), stratified by sex.

	**Women**			**Men**		
	**RR**	**95% CI**	p-value	**RR**	**95% CI**	p-value
Pre-post COVID-19	1.17	1.14; 1.20	<0.001	1.15	1.11; 1.18	<0.001
Age	1	1.00; 1.00	<0.001	1.01	1.01; 1.01	<0.001
Socioeconomic index	0.99	0.99; 0.99	<0.001	0.99	0.99; 0.99	<0.001

RR = rate ratio; CI = confidence interval.

Table S2: Association between Socioeconomic Index quartiles and waiting time for ophthalmology surgery before and after the COVID-19 pandemic, stratified by sex.

	**Women**					
	**Pre-pandemia**			**Post-Pandemia**		
	**RR**	**95% CI**	p-value	**RR**	**RR**	p-value
**Age**	1	1.00; 1.01	<0.001	1	1.00; 1.01	0.012
**Socioeconomic Index**						
Low	1.33	1.26; 1.40	<0.001	1.18	1.11; 1.26	<0.001
Middle to Low	1.08	1.03; 1.14	<0.001	1.06	0.99; 1.12	0.077
Middle to high	1.02	0.97; 1.08	0.015	1.07	1.01; 1.14	0.020
High (ref.)	--	--	--	--	--	--
	**Men**					
	**Pre-pandemia**			**Post-Pandemia**		
	**RR**	**95% CI**	p-value	**RR**	**95% CI**	p-value
**Age**	1.01	1,01; 1,01	<0.001	1.01	1.01; 1.01	<0.001
**Socioeconomic Index**						
Low	1.37	1.28; 1.47	<0.001	1.21	1.12; 1.32	<0.001
Middle to Low	1.15	1.08; 1.22	<0.001	1.11	1.03; 1.20	0.007
Middle to high	1.08	1.02; 1.15	0.015	1.1	1.02; 1.18	0.016
High (ref.)	--	--	--	--	--	--

RR = rate ratio; CI = confidence interval.
